# Molecular Beam Scattering Experiments as a Sensitive Probe of the Interaction in Bromine–Noble Gas Complexes

**DOI:** 10.3389/fchem.2019.00320

**Published:** 2019-05-17

**Authors:** David Cappelletti, Antonio Cinti, Andrea Nicoziani, Stefano Falcinelli, Fernando Pirani

**Affiliations:** ^1^Dipartimento di Chimica, Biologia e Biotecnologie, Università degli Studi di Perugia, Perugia, Italy; ^2^Dipartimento di Ingegneria Civile ed Ambientale, Università degli Studi di Perugia, Perugia, Italy

**Keywords:** halogen bond, charge transfer, molecular beam scattering, bromine, noble gases

## Abstract

This paper reports for the first time molecular beam experiments for the scattering of He, Ne, and Ar by the Br_2_ molecule, with the aim of probing in detail the intermolecular interaction. Measurements have been performed under the experimental condition to resolve the glory pattern, a quantum interference effect observable in the collision velocity dependence of the integral cross section. We analyzed the experimental data with a reliable potential model defined as a combination of an anisotropic van der Waals component with the additional contribution due to charge transfer and polar flattening effects related to the formation of an intermolecular halogen bond. The model involves few parameters, whose values are related to fundamental physical properties of the interacting partners, and it allows an internally consistent comparison of the stability of the gas-phase adducts formed by Br_2_ moiety with different noble gases as well as homologous complexes with the Cl_2_ molecule. The same model appears to be also easily generalized to describe the interaction of diatomic halogen molecules in the excited B(^3^Π) electronic state where the halogen bond contribution tends to vanish and more anisotropic van der Waals components dominate the structure of the complexes with noble gases.

## Introduction

The knowledge of the nature and the characterization of the role of the intermolecular halogen bond (XB) is presently recognized to be of great relevance in many areas of fundamental and applied research, including materials engineering, biochemistry, molecular recognition, drug design, and supra-molecular Chemistry (Gilday et al., 2015; Han et al., 2017).

In order to disentangle the effect of the XB on the molecular dynamics, it is necessary to identify the interaction components involved and to provide their radial and angular dependences. This information, seldom available in the literature, can obtained by investigating in detail prototypical systems, whose features are necessary to formulate interaction models useful for the description of the force fields in systems at increasing complexity and of applied interest (Cappelletti et al., 2015).

The weakly bound complexes Ng–X_2_, formed by a noble gas (Ng) and a di-halogen molecule X_2_ (X=Cl, Br, I), have been considered as prototypes of particular relevance for investigating energy transfer mechanisms and for the characterization of the fundamental role of the intermolecular interaction components (Baturo et al., 2017; Li et al., 2017) leading to the formation of the weak intermolecular halogen bond (Desiraju et al., 2013). Moreover, for the identification of basic selectivity in energy transfer processes, the X_2_ moiety has been considered both the ground ($X^{2}\Sigma_{g}^{+}$) and in the excited ($B^{3}\Pi_{u}$) electronic state (Janda et al., 1998; Rohrbacher et al., 2000; Delgado-Barrio et al., 2006; Beswick et al., 2012), and related potential energy surfaces (PES) have been classified as *X* (ground) and *B* (excited).

Extensive spectroscopic and theoretical studies (see, for instance, Jahn et al., 1996; Buchachenko et al., 2000; Prosmiti et al., 2002a,b; de Lara-Castells et al., 2004; Boucher et al., 2005; Garcia-Vela, 2005; Carrillo-Bohórquez et al., 2016) have been devoted to the characterization of the stability of the Ng–Br_2_ adducts in the limiting collinear and T-shaped configurations and of the predissociation dynamics induced by electronic, vibrational, and rotational excitations.

Nowadays, it is clear that in the ground-state PES, both the collinear and T-shaped isomers have comparable binding energy and are separated by a significant saddle region. By contrast, in the electronically excited PES, the T-shaped configuration is the most stable one, as typical of most of the atom-diatom complexes bound by van der Waals (vdW) forces (de Lara-Castells et al., 2004; Garcia-Vela, 2005; Pirani et al., 2019). The origin of this difference in the topography of the PES is still being debated because it depends on a delicate balance between the involved interaction components. In particular, open questions concern the proper identification of the principal interaction terms, their modeling, and their dependence on the atomic or molecular partners involved within the complex.

Recently, we performed an integrated experimental/theoretical investigation on Ng–Cl_2_ systems with the goal of adequately addressing some of the above-mentioned open questions (Nunzi et al., 2019; Pirani et al., 2019). In particular, we have found that for such systems, the most relevant features of the *X* ground-state PESs are mainly determined by the anisotropic halogen bond components, which operate even in the case of the lightest He–Cl_2_. Such components concur to stabilize the collinear configuration selectively by charge transfer (CT) and polar flattening (PF) effects, which are specific interaction features of XB. *Ab initio* calculations have revealed that both CT and PF contributions miss in the electronic excited *B* PESs, where the formed adducts show typical vdW behavior. This observation is consistent with the behavior of other atom–diatom systems, as Ng–O_2_ and Ng–N_2_ complexes, dominated by *size repulsion* and *dispersion/induction attraction* (Aquilanti et al., 1995).

The present manuscript reports and discusses the results of a new experimental investigation, focused on the *X* ground PES of the He–, Ne–, and Ar–Br_2_ systems, carried out with the molecular beam (MB) technique applied under the same conditions of recent experiments on Ng–Cl_2_. The analysis of these new scattering data has been performed by extending the methodology applied to the rationalization of data measured for homologous systems formed with the Cl_2_ moiety (Nunzi et al., 2019; Pirani et al., 2019). In particular, the main components, characterizing the interaction potential between Ng and Br_2_, have been identified and their radial angular dependences represented through the adoption of semi-empirical/empirical equations involving only a few parameters, each one with a defined physical meaning. As for Ng–Cl_2_ systems, we have found that *X* ground-state PES in Ng–Br_2_ adducts is mainly determined by anisotropic halogen bond components, concurring to stabilize selectively the collinear configuration by CT and PF effects. The model has also been applied to predict the behavior of Kr– and Xe–Br_2_ systems, for which MB experiments cannot be carried out under sufficiently high angular and velocity resolution conditions, obtainable for lighter Ng atoms, which are proper to resolve quantum interference effects in the scattering. Such an extension has been achieved by simply exploiting the change of parameters involved when one is moving along the Ng–Br_2_ homologous family of systems. Finally, the features the obtained PES have been compared with results from the literature.

## Experimental Apparatus and Scattering Results

Gas-phase scattering experiments have been carried out in order to measure the velocity dependence of the integral cross section. The availability of the projectile (here, He, Ne, and Ar) in a large speed range is of great relevance to perform measurements as a function of the collision velocity. On the other hand, the choice of temperature and pressure of the target with a defined mass [here, Br_2_ (*X*,^1^$\Sigma_{\text{g}}^{+}$)] is crucial to achieve in the experiments angular and velocity resolution conditions proper to resolve quantum interference effects, as those giving the “glory” oscillations, observable in the velocity dependence of the integral cross section. The collected experimental results probe in detail, and an internally consistent way, the absolute scale of the interaction both at long and intermediate distance ranges, where, respectively, the attraction dominates and the potential well occurs (Pirani and Vecchiocattivi, 1982; Pirani et al., 2008). Therefore, such results provide direct information on some basic features of the *X* PESs and allow a direct comparison with other *X* PESs, recently characterized in detail for the corresponding Ng–Cl_2_ systems (Nunzi et al., 2019; Pirani et al., 2019).

The experiments have been performed with an MB apparatus, with the objective of measuring the total (elastic + inelastic) integral cross section *Q* as a function of the selected MB velocity *v*. Such an apparatus has been extensively described in the past (Aquilanti et al., 1992; Cappelletti et al., 2002, 2015, 2016a,b). Briefly, it is composed of a set of differentially pumped vacuum chambers, where MB, in the present case formed by Ng atoms, is generated by the gas expansion from a nozzle, maintaining its temperature in the range of 77–600 K and total pressure in the source within 7–20 mbar, in order to avoid cluster formation and to cover a wide range of collision velocities. Under such conditions, the MB emerges with near-effusive or moderate supersonic character, and it is analyzed in velocity by a mechanical selector and collides at a “nominal” velocity, *v*, with the stationary target gas (Br_2_) contained in the scattering chamber at a pressure not larger than 2 × 10^−4^ mbar in order to assure the occurrence of single collision events. The chamber is kept at room temperature to avoid condensation effects of the target gas on the walls and to maintain a sufficiently high rotational temperature of the target molecules. The latter condition is critical to limit anisotropy effects in the scattering and then to better resolve frequency and amplitude of the glory oscillatory pattern. MB is detected downstream by a quadrupole mass spectrometer, coupled with an ion counting device. At each selected velocity, *v*, of the projectile atoms, the quantity to be measured is the MB attenuation *I/I*_0_, where *I* represents the MB intensity detected with the target in the scattering chamber (filled at the chosen pressure) and *I*_0_ that without it (empty chamber). From the measurement of the ratio *I/I*_0_, it is possible to determine the value of the integral cross section *Q(v)* through the Lambert–Beer law: calibration methodology and reference data are given in Nenner et al. (1975), Aquilanti et al. (1976), Pirani and Vecchiocattivi (1977).

**Figure 1:**
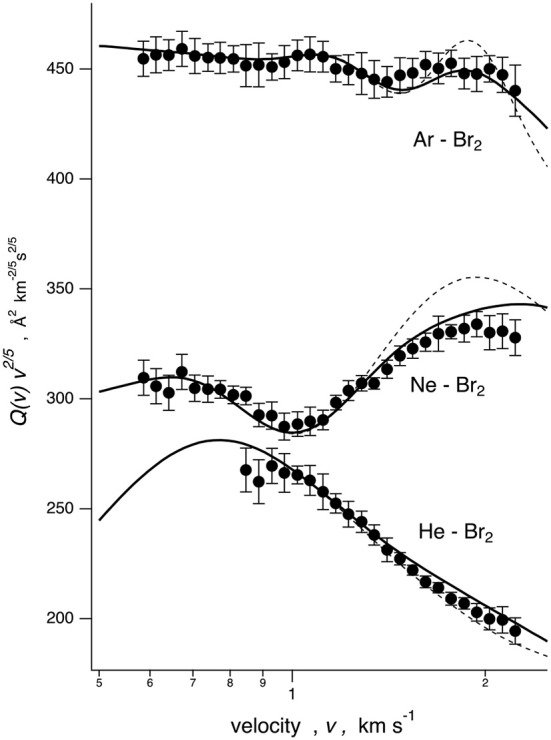
Integral cross sections *Q(v)* for Ng atom projectiles colliding at each selector velocity with Br_2_ targets. Data are plotted as *Q(v)*· *v*^2/5^ to emphasize the glory patterns. Solid line: cross sections calculated with the full PES, including in the formulation of the various interaction components taken into account in the data analysis. Dashed line: calculation with the spherically averaged PES.

The *Q(v)* values, measured for He–, Ne–, and Ar–Br_2_ systems as a function of the selected MB velocity *v*, are reported in Figure 1. In all cases, the cross sections are plotted as *Q(v)*· *v*^2/5^ to emphasize the “glory” quantum interference and to more efficiently analyze the scattering cross sections in terms of a smooth component and an oscillating part. The He–, Ne–, and Ar–Br_2_ systems exhibit absolute scales of the observables and interference patterns that are very different, thus revealing significant variations in the intermolecular interactions.

The analysis of *Q(v)* (see next sections) provided a quantitative characterization of the strength of the intermolecular interaction both at long range, obtained from the velocity dependence of the average value of *Q(v)*, and in the potential well region, probed by the resolved glory structure (Pirani and Vecchiocattivi, 1982; Pirani et al., 2008).

During the analysis, center-of-mass (CM) cross section values have been calculated within the semi-classical Jeffreys–Wentzel–Kramers–Brillouin approximation (Child, 1974) from the assumed intermolecular interaction potential *V* (see next section), and afterwards convoluted in the laboratory frame to make a direct comparison with the measured *Q(v)* (Cappelletti et al., 2002).

During a trial-and-error procedure, the parameters defining the basic features of *V* have been tested and fine-tuned in order to obtain the best comparison between experimental and calculated data. This *phenomenological* analysis (see the next section) has been guided also by available results obtained in the past on a large variety of atom–molecule systems (Pirani et al., 2019).

## Potential Parametrization and Data Analysis

For the Ng–Br_2_ systems, we adopted a formulation of PES based on what recently developed for the homologous systems with Cl_2_ (Nunzi et al., 2019). In particular, the total intermolecular potential *V* has been defined as the sum of three contributions, identified as vdW, *V*_*vdW*_, three bodies, *V*_3*B*_, and CT, *V*_*CT*_, each one related to fundamental features of the partners involved in the interaction.

The electronic polarizability is the fundamental chemical–physical property determining both dispersion/induction attraction and Pauli (exchange or particle size) repulsion and can be employed in semi-empirical correlation formulas (Cambi et al., 1991) for the modeling of a large variety of non-covalent intermolecular interactions. Accordingly, the anisotropic *V*_*vdW*_ component has been represented in terms of *two* pairwise additive potential contributions, Ng–Br_*i*_, where the Br_*i*_ interaction centers coincide with the bromine atoms of the Br_2_ molecule. As emphasized for the representation of Cl atoms in Cl_2_ (Nunzi et al., 2019), also for these “effective” Br atoms, involved in a stable Br–Br chemical bond, we assumed an anisotropic component of the electronic polarizability different from that of the isolated Br atom. On the other side, the value obtained by summing the average polarizability of the “effective” Br atoms is kept consistent with that of the Br_2_ molecule (Maroulis and Makris, 1997). To adequately describe the repulsion contributions related to the strongly anisotropic Br_2_ electron density, mostly determined by the outer valence electrons in the π^*^ molecular orbitals, we included in the formulation of *V* a three-body term, *V*_3B_. Finally, a third interaction component, *V*_CT_, has been included and associated with CT effects, which directly influences the formation of the intermolecular bond (Pirani et al., 2000; Belpassi et al., 2009; Cappelletti et al., 2012).

**Figure 2:**
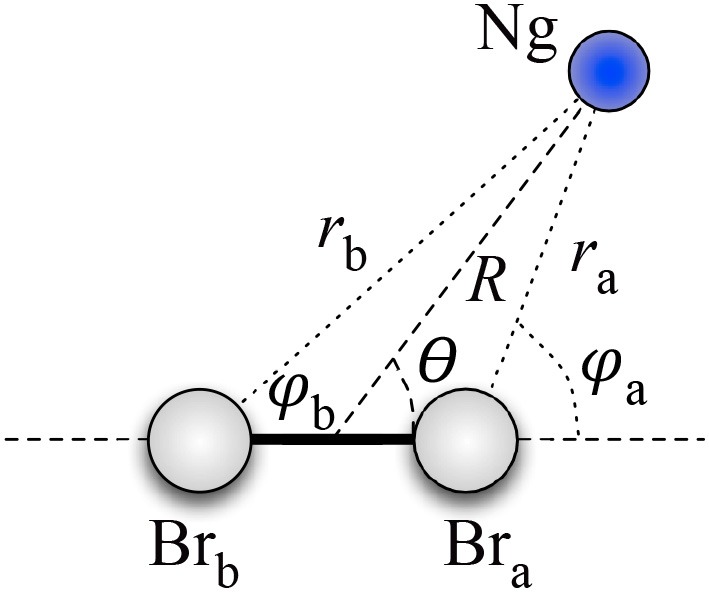
Coordinate systems for the Ng–Br_2_ systems under study.

Therefore, assuming as *R* the distance between Ng and the CM of Br_2_, and as θ the angle between the vector **R** and the Br–Br bond axis (see Figure 2), for the Ng–Br_2_ adducts in the *X* ground states, we defined the anisotropic intermolecular potential *V(R*, θ*)* as the combination of three main components:

(1) $$\begin{array}{r} V\left(\;R,\theta \right)=V_{vdW}\left(R,\theta \right)+V_{CT}\left(R,\theta \right)+V_{3B}\left(R,\theta \right) \end{array}$$

Such components indirectly include the role of less important contributions.

In more detail, *V*_*vdW*_ has been represented as the sum of two Ng–Br_*i*_ (*i*= *a, b*) pairwise additive contributions:

(2) $$\begin{array}{r} V_{vdW}\left(R,\theta \right)=V_{Ng-Br_{a}}\left(r_{a},\varphi_{a}\right)+V_{Ng-Br_{b}}\left(r_{b},\varphi_{b}\right) \end{array}$$

where *a* and *b* identify the two different Br atoms, *r*_*a*_ and *r*_*b*_ are the distances between Ng and Br_*a*_/Br_*b*_, and ϕ_*a*_and ϕ_*b*_ are the angles between **r**_**a**_**/r**_**b**_ and the Br_2_ bond axis. Accordingly, each atom–atom pair term has been formulated as an improved Lennard Jones (ILJ) function (Pirani et al., 2008):

(3) $$\begin{array}{l} V_{Ng-Bri}\left(r_{i},\varphi_{i}\right)=\varepsilon (\varphi_{i})[\frac{6}{n\left(r_{i},\varphi_{i}\right)-6}\cdot {\left(\frac{r_{m}(\varphi_{i})}{r_{i}}\right)}^{n\left(r_{i},\;\varphi_{i}\right)}\\ \;-\frac{n(r_{i},\varphi_{i})}{n\left(r_{i},\varphi_{i}\right)-6}\cdot {\left(\frac{r_{m}(\varphi_{i})}{r_{i}}\right)}^{6}] \end{array}$$

where the ε(φ_*i*_) and *r*_*m*_(φ_*i*_) parameters are generated by the following relationships:

(4) $$\begin{array}{r} \varepsilon \left(\varphi_{i}\right)\;=\;\varepsilon_{∥}\cdot \cos^{2}\left(\varphi_{i}\right)+\varepsilon_{⊥}\cdot \sin^{2}\left(\varphi_{i}\right) \end{array}$$

(5) $$\begin{array}{r} r_{m}\left(\varphi_{i}\right)\;=\;{r_{m}}_{∥}\cdot \cos^{2}\left(\varphi_{i}\right)+{r_{m}}_{⊥}\cdot \sin^{2}\left(\varphi_{i}\right) \end{array}$$

The symbols ∥ and ⊥ refer, respectively, to the parallel (φ_*i*_ = 0) and perpendicular (φ_*i*_ = π*/2*) configurations within each Ng–Br_*i*_ pair. The factor *n(r*_*i*_, φ_*i*_*)*, which modulates simultaneously the “fall off” of the repulsion and the radial dependence of the intermediate and long-range attraction, depends on β, an additional parameter related to the hardness of both partners (Capitelli et al., 2007). It is expressed as,

(6) $$\begin{array}{r} n\left(r_{i},\varphi_{i}\right)=\;\beta +4\cdot {\left(\frac{r_{i}}{r_{m}\left(\varphi_{i}\right)}\right)}^{2} \end{array}$$

Note that the partial long-range attraction coefficient, *C*_6*i*_ = $\varepsilon \left(\varphi_{i}\right)\cdot r_{m}^{6}\left(\varphi_{i}\right),$ provides the asymptotic behavior of each ILJ contribution, while the global attraction coefficient is simply given as the sum of the two angular averaged *C*_6*i*_ components.

The values of the ε and *r*_*m*_ parameters have been predicted in an internally consistent way for the three Ng–Br_2_ systems from the polarizability components (Cambi et al., 1991). They have been tested, and when necessary, fine-tuned, by exploiting the comparison of calculated cross sections with experimental results. During the analysis, the additional constraint of providing global average asymptotic attractions in substantial agreement (within about 10%) with those reported by Olney et al. (1997) has been imposed. As previously done for other systems involving other halogen atoms (Bartocci et al., 2015; Cappelletti et al., 2015; Nunzi et al., 2019), the zero-order values of _*r*_*m*_ ∥_ have been decreased by about 4% to account for the PF effect in the *X* ground state of Br_2_. Such an effect must be related to the peculiar electronic charge distribution of Br along the Br–Br bond direction pointing at the approaching Ng in the collinear isomer (see next section). The decreasing of _*r*_*m*_ ∥_ has been accompanied by an of *e*_∥_ in order to maintain the *C*_6_ coefficient constant.

The second term in Equation 1, *V*_3*B*_*(R*, θ*)*, has been formulated as,

(7) $$\begin{array}{r} V_{3B}\left(R,\theta \right)=-A_{3B}{\left(sin2\theta \right)}^{2}\cdot e^{-3.0\cdot R} \end{array}$$

and it has been enclosed to properly represent the angular dependence of the full PES, especially in the proximity of the saddle point, where the molecular repulsion by occupied π^*^ orbitals is more prominent. The functional form of V_3B_ has been improved with respect to that reported by Nunzi et al. (2019) to guarantee a second derivative equal to zero at θ = *90*.

Finally, the third term, *V*_*CT*_*(R*,θ*)*, has been defined as,

(8) $$\begin{array}{r} V_{CT}\left(R,\theta \right)=\underset{i=a,b}{\sum }A_{CT}\cdot \cos^{4}\left(\varphi_{i}\right)\cdot e^{-3.0\cdot r_{i}} \end{array}$$

The dynamical treatment used for the data analysis, adopted for many other atom–molecule systems and summarized in the Supporting Information (SI), allows a good reproduction of the measured cross sections for all the investigated systems by mostly adjusting *A*_3B_ and *A*_CT_ values, while keeping unaltered (or variable in limited ranges) the other parameters. The final values are reported in Table 1.

**Table 1:** Potential parameters (ε, *A*_CT_, *A*_3B_ in meV, and *r*_m_ in Å) employed for the formulation of the Ng–Br atom–atom pairwise interaction for Ng–Br_2_ systems in the (${\text{X}}^{1}\Sigma_{\text{g}}^{+}$) ground and (${\text{B}}^{3}\Pi_{0\text{u}+}$) excited states.

	**AA pair**	***r*_**m||**_**	**ε||**	***r*_**m**_⊥**	***ε*⊥**	***A*_**CT**_ × 10^**5**^**	***A*_**3B**_ × 10^**5**^**
	He–Br	3.53	4.71	3.71	3.02	0.44	6.2
	Ne–Br	3.57	9.66	3.75	6.21	1.22	9.0
Br_2_(${\text{X}}^{1}\Sigma_{\text{g}}^{+}$)^(a)^	Ar–Br	3.80	21.3	4.00	13.9	4.40	14.0
	Kr–Br	*3.87*	*28.9*	*4.07*	*18.4*	*9.00*	*16.6*
	Xe–Br	*4.01*	*34.5*	*4.23*	*21.9*	*17.5*	*19.0*
	He–Br	*3.83*	*2.92*	*3.81*	*2.66*		
	Ne–Br	*3.83*	*6.27*	*3.83*	*5.53*		
Br_2_(${\text{B}}^{3}\Pi_{0\text{u}}^{+}$)^(b)^	Ar–Br	*3.96*	*17.7*	*4.02*	*14.4*		
	Kr–Br	*4.04*	*23.3*	*4.11*	*18.6*		
	Xe–Br	*4.16*	*29.6*	*4.26*	*22.9*		

*Data for He–, Ne–, and Ar–Br_2_(X) have been determined from the experimental best fit; the others (in italics) are from model calculations (see text)*.

(a) *The β parameter of the ILJ function (see text) has been fixed to 7.0 for all atom–atom pairs. The maximum estimated uncertainty is about 5% for ε, 2% for r_m_, and 15% for A_CT_ and A_3B_*.

(b) *The β parameter of the ILJ function (see text) has been fixed to 7.0 for all atom–atom pairs. The maximum estimated uncertainty is about 10% for ε and 3% for r_m_*.

## Discussion

**Figure 3:**
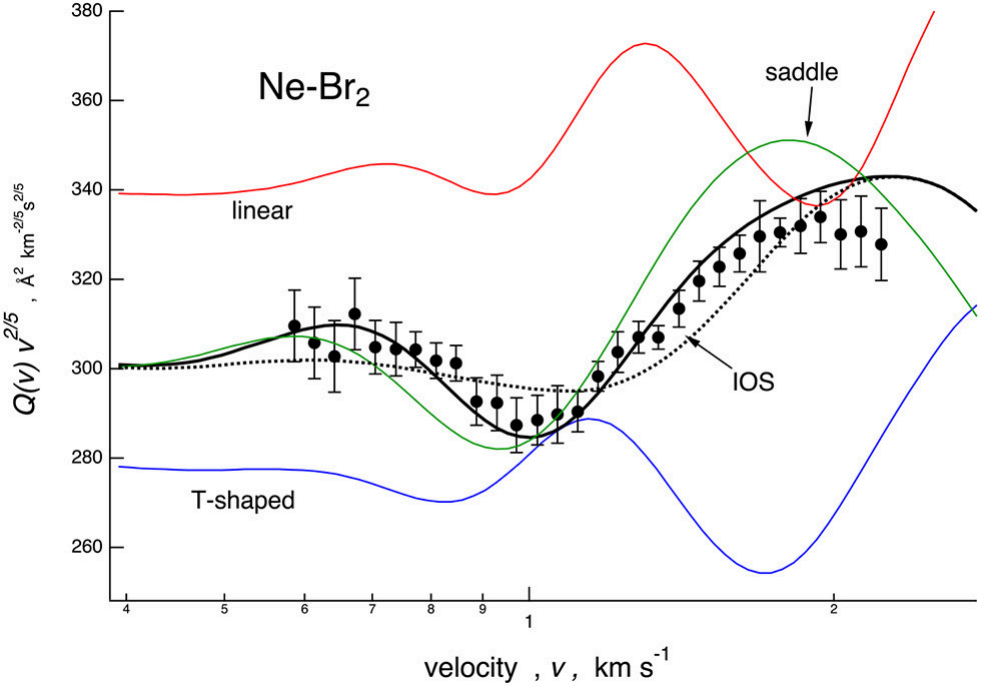
Comparison for Ne–Br_2_ system between experimental cross section data, plotted as *Q(v)*· *v*^2/5^ and reported as a function of the molecular beam (MB) velocity *v*, and calculations performed considering the interaction in the three selected limiting configurations (colored lines), the infinite-order sudden (IOS) approximation (dotted line), and the full treatment employed for the data analysis (solid line, see text).

**Figure 4:**
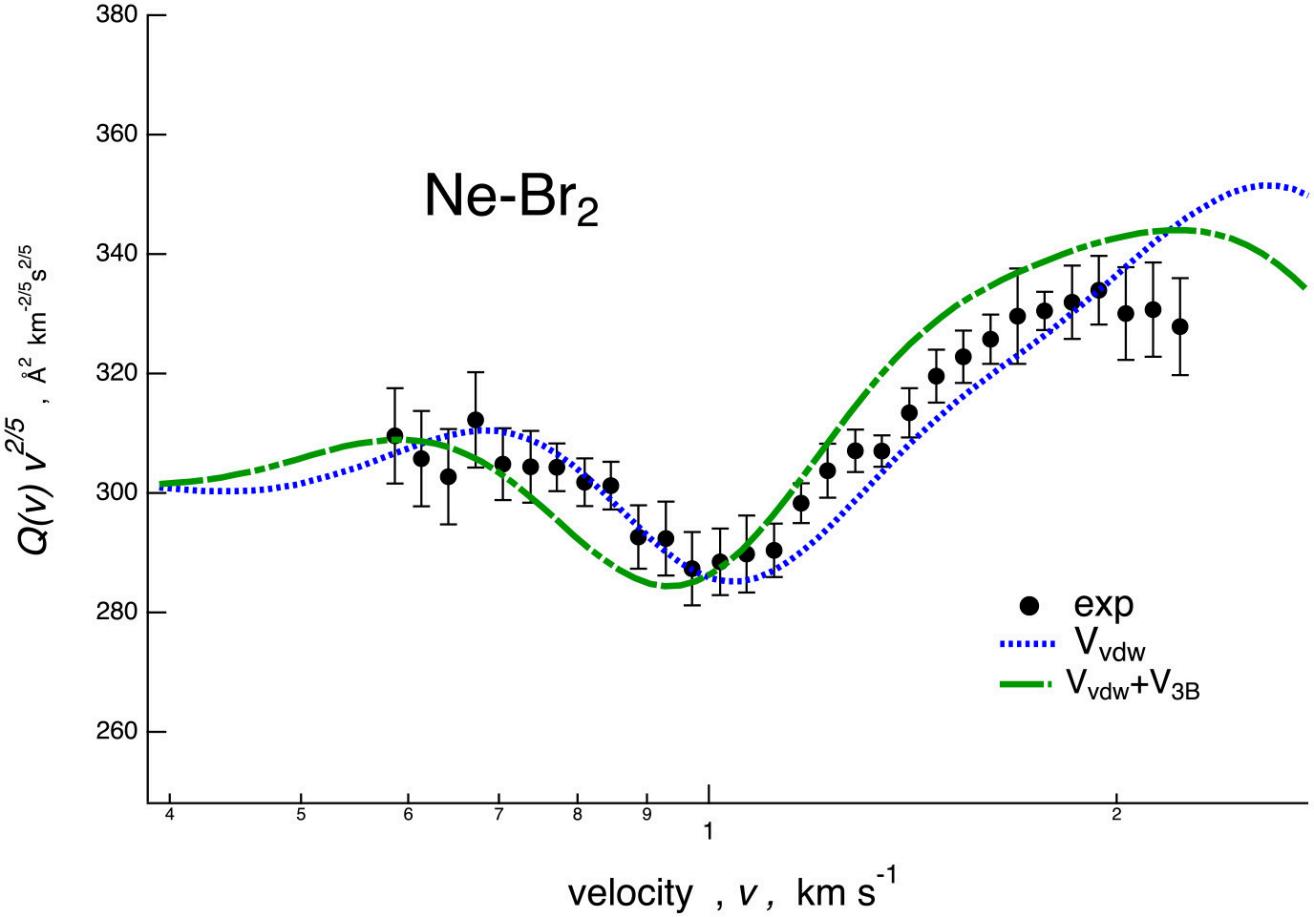
Comparison for Ne–Br_2_ system between experimental cross section data, plotted as *Q(v)*· *v*^2/5^ and reported as a function of the MB velocity *v*, and calculations performed considering a potential energy surface (PES) including only the van der Waals component plus PF contribution (V_vdW_, dotted line) and the additional three-body contribution (V_vdW_+V_3B_, dashed line).

Cross sections, calculated with the full PES based on the potential parameters of Table 1, are compared with the experimental data in Figure 1. In the same figure is also reported, as a dashed line, a calculation based on the spherically averaged PES. In Figures 3, 4, some details are given on the dynamical model employed and on the sensitivity of the experiment to the PES features. Results are presented for the case of Ne–Br_2_, chosen as a representative. Specifically in Figure 3, scattering cross sections derived from selected cuts of the PESs are reported together with those obtained combining them according to those obtained according to the IOS (infinite-order sudden) approximation and to the dynamical regime adopted, whose details are reported by Nunzi et al. (2019) and also summarized in Supporting Information.

The IOS calculations are very sensitive to the anisotropy of the PES and must be considered correct to describe the scattering when the collisions are “sudden”: this typically occurs at high relative collision velocities. The data treatment exploited in the present work combines the IOS results with cross sections calculated with the spherically averaged PES, probed in the present investigation by collisions confined at low velocities (see Figure 1) by means of a switching function operative in the intermediate velocity range (see the Supplementary Information). Therefore, this treatment exploits the concept that present observables are basically determined by anisotropic elastic collisions and that inelastic events, occurring at orbital angular momentum values smaller than those probed by the present experiments, play a minor role (Aquilanti et al., 1998). The combined calculations reproduce the amplitude satisfactorily and the frequency of the glory patterns very well, experimentally resolved for all investigated systems, and this represents an important reliability test for all proposed PESs. They have been formulated in an internally consistent way and using few parameters, all related to basic physical properties of the interacting partners. In particular, the parameters that define *V*_*vdW*_ depend on the polarizability components of Br_2_ and scale also according to that of Ng, while the significant effect of *V*_3*B*_manifests along the direction of π^*^ molecular orbitals, occupied by more outer electrons of Br_2_.

In Figure 4, we report the results of a sensitivity test for the Ne–Br_2_ case. In particular, a calculation has been performed with a PES including the term and the PF effect (*V*_vdW_, blue dotted) and further adding the three-body term (*V*_vdW_ + *V*_3B_, green dashed). These incomplete PESs fail in reproducing the correct location of the calculated glory interference extrema.

The average strength of *V*_*CT*_ increases from He to Ar, accordingly with the ionization potential of Ng. It must also depend on the electron affinity of Br_2_, which is slightly larger with respect to that of Cl_2_. Moreover, its angular dependence is strongly modulated by the PF and by the so-called σ-hole in the electron density of the halogen molecule (Clark et al., 2007; Kol and Hobza, 2016).

**Figure 5:**
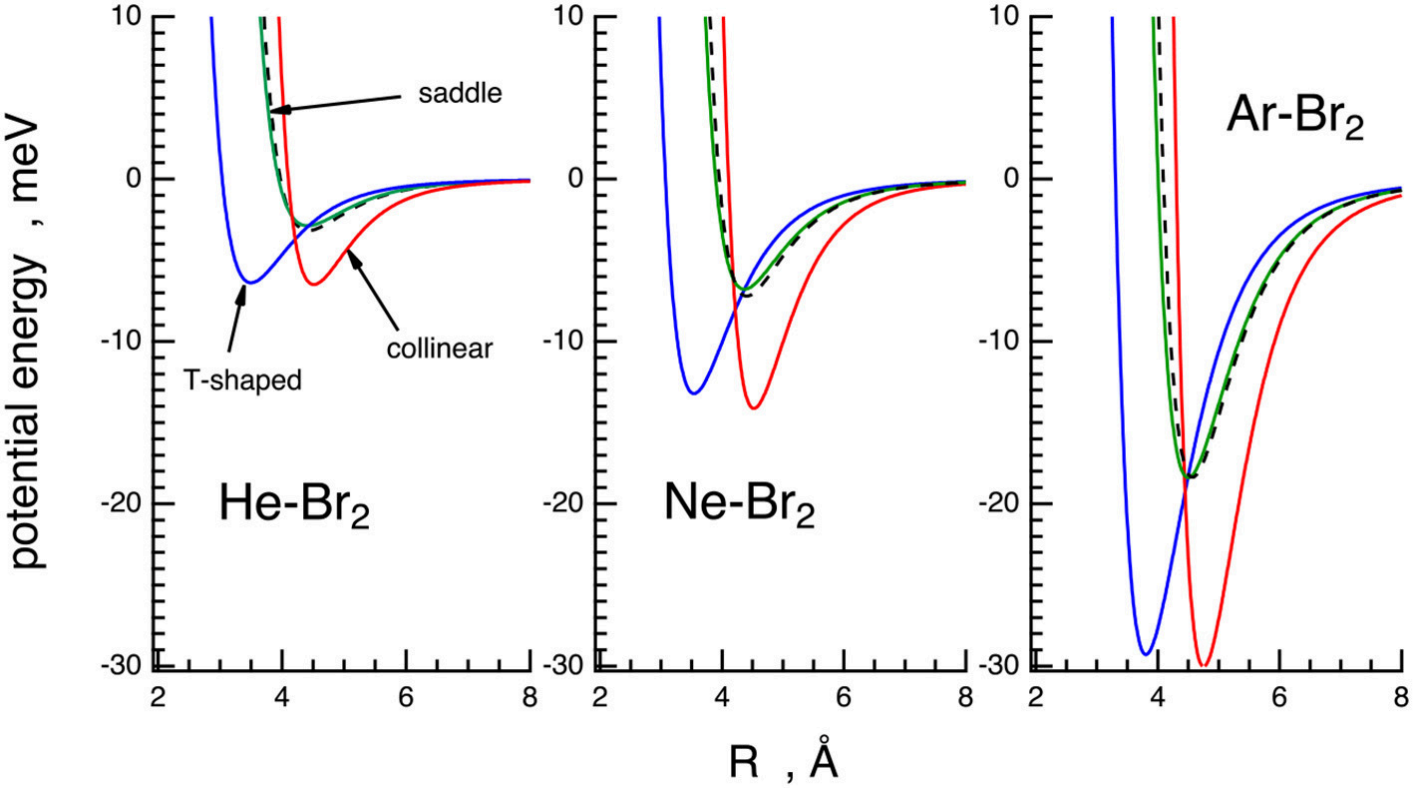
Potential energy curves (energy vs. Ng–Br_2_ distance) of the X ground state for the Ng–Br_2_ complexes in the three selected configurations obtained from the phenomenological PESs. T-shaped (θ = *90*, blue solid line), collinear (θ = *0*, red solid line), saddle (θ values in Table 2, green solid line). The spherically averaged interactions are reported for comparison (black dashed lines).

In order to illustrate the main basic features of the PESs characterized in this paper, the interaction energy for selected configurations of Ng–Br_2_ systems is plotted as a function of the Ng–Br_2_ distance, *R*, in Figure 5.

These results can be compared with those obtained for the analogous Ng–Cl_2_ systems recently (Nunzi et al., 2019; Pirani et al., 2019). When passing from Cl_2_ to Br_2_ adducts, the relative anisotropy, obtained by scaling the absolute anisotropy for the average interaction, is very similar, but the absolute interaction is higher for the Br_2_ family. The variation can be attributed to the simultaneous increase of all the basic components, here represented as *V*_*vdW*_, *V*_3*B*_, and *V*_*CT*_, due to the combined change of electronic polarizability and of the extension of external charge distribution when moving from Cl_2_ to Br_2_.

In Table 2, the main features of the obtained PESs, namely, binding energy and equilibrium distance for the three basic configurations of each system, are given for the noble gas–Br_2_(X) systems. In the table are also reported significant results from the literature, mostly from *ab initio* calculations, available for the He, Ne, and Ar cases.

**Table 2:** Potential well depth (ϵ, in meV) and well location r_m_ (in Å) for the Ng–Br_2_ complexes for the (${\text{X}}^{1}\Sigma_{\text{g}}^{+}$) ground and (${\text{B}}^{3}\Pi_{0\text{u}+}$) excited states of Br_2_ representative of the intermolecular bond stability and length in the three basic configurations.

		***T-shaped***		***Collinear***		***Saddle***		
	***r_***m***_***	**ε**	***r_***m***_***	**ε**	***r_***m***_***	**ε**	**θ**	
He–Br_2_(X)	3.50	6.4	4.51	6.5	4.37	2.8	59	present exp.
	*3.58*	*5.0*	*4.42*	*6.1*				(Prosmiti et al., 2002a,b)
	*3.55*	*5.6*	*4.42*	*6.0*	*4.5*	*2.3*	*51*	(Valdes et al., 2004)
	*3.6*	*5.0*	*4.41*	*6.1*				(Boucher et al., 2005)
Ne–Br_2_(X)	3.54	13.2	4.52	14.1	4.33	6.9	57	present exp.
	*3.60*	*10.7*	*4.49*	*11.6*				(Prosmiti et al., 2002a,b)
Ar–Br_2_(X)	3.81	29.3	4.75	30.0	4.52	18.2	54	present exp.
	*3.80*	*28.1*	*4.63*	*32.6*				(Prosmiti et al., 2002a,b)
Kr–Br_2_(X)	*3.89*	*38.8*	*4.77*	*43.9*	*4.57*	*25.4*	*54*	present model
Xe–Br_2_(X)	*4.04*	*46.2*	*4.86*	*55.0*	*4.72*	*31.5*	*53*	present model
He–Br_2_(B)	*3.61*	*5.4*	*5.03*	*3.2*				present model
	*3.65*	*4.2*	*5.29*	*2.1*				(Garcia-Vela, 2005)
	3.7^*^	4.1^*^	*5.17*	*2.6*				(de Lara-Castells et al., 2004)
Ne–Br_2_(B)	*3.63*	*11.2*	*5.03*	*6.8*				present model
Ar–Br_2_(B)	*3.82*	*29.4*	*5.16*	*19.2*				present model
Kr–Br_2_(B)	*3.92*	*38.0*	*5.24*	*25.4*				present model
Xe–Br_2_(B)	*4.07*	*46.9*	*5.35*	*32.5*				present model

* *Average of A′ and A″ PES*.

*Data for He–, Ne–, and Ar–Br_2_(X) have been determined from the experimental best fit, the others (in italics) from a semi-empirical model or ab initio calculations (see text)*.

Within the same approach, we have also predicted the interaction parameters for Kr–Br_2_ and Xe–Br_2_ systems, whose values have been enclosed in Tables 1, 2. Specifically, the vdW *r*_m_ , ε, and the *A*_3B_ parameters of Kr– and Xe– cases (Table 1) have been obtained by a direct scaling of those of the lighter rare gases' parameters, utilizing the noble gas polarizabilities and correlation formulas of general validity (Cambi et al., 1991). The CT parameter A_CT_ has been obtained through correlation formulas for the CT component (see Pirani et al., 2000 and references therein), utilizing the ionization potential of the noble gases and the electron affinity of the halogen molecule (Pirani et al., 2000).

The agreement with literature data is, in general, very good. The present results for the He–Br_2_ and Ne–Br_2_ cases show slightly deeper well depths.

**Figure 6:**
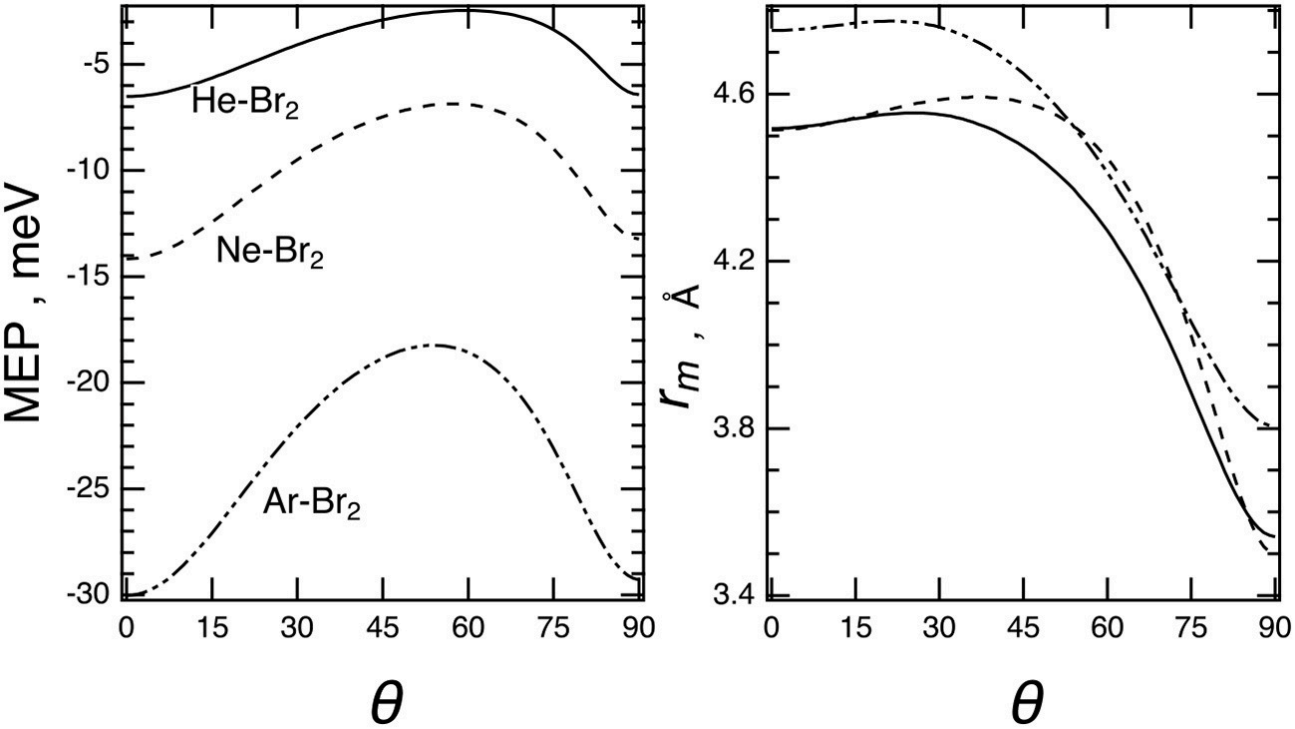
Minimum energy path, *MEP* (left), and equilibrium distances, *r*_m_ (right), vs. the angular variable θ for the Ng–Br_2_ complexes (Ng = He, Ne, Ar) predicted by the present potential formulation for the ground (X) electronic states of Br_2_.

The minimum energy path, *MEP*, describing the angular dependence of the binding energy evaluated at each equilibrium distance is given in Figure 6. The *MEP* has been easily obtained here by exploiting the adopted analytical formulation of the interaction.

**Figure 7:**
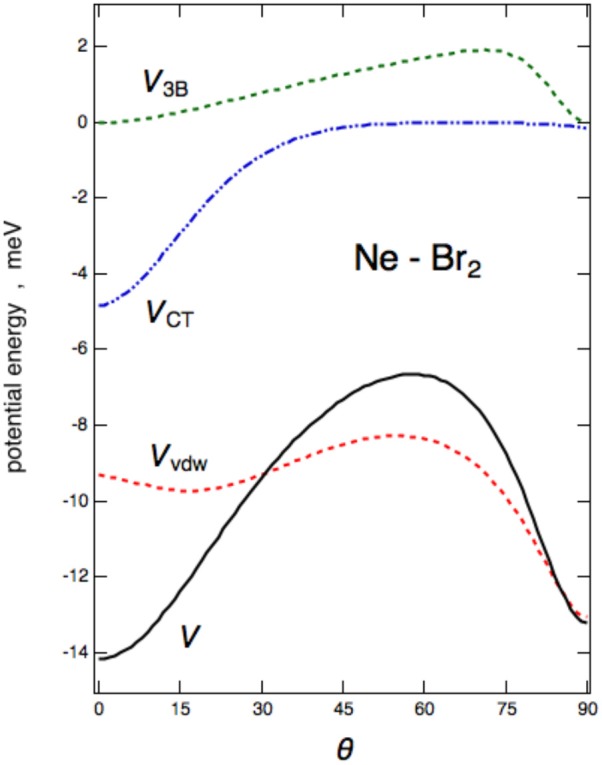
Interaction energies for the *V*_*vdW*_, *V*_3*B*_, and *V*_*CT*_ components vs. the angular variable θ are reported for the Ne–Br_2_(X) case.

The angular trend of the interaction component *V*_*vdW*_, *V*_3*B*_, and *V*_*CT*_, is reported for Ne–Br_2_ in Figure 7. From the figure, it can be clearly seen that *V*_*vdW*_ alone would provide a T-shaped configuration (θ=90°) much more stable than the collinear (θ=0°); on the other side, the *V*_*CT*_ term is responsible for the change in stability of the collinear configuration with respect to the perpendicular one. The effect of the *V*_3*B*_ term manifests itself at an intermediate angle and affects mostly the relative stability of the saddle configuration.

The phenomenological approach has also been extended to obtain, within the same framework adopted for the systems involving Cl_2_, an analytical formulation of the PESs for the complete family of the Ng(^1^S_0_)–Br_2_($B^{3}\Pi_{0\text{u}+}$) systems. Upon excitation in the triplet B state, leading an electron from the π^*^ to the σ^*^ molecular orbital, a consistent charge rearrangement is attained in the Br_2_ molecule, accomplishing an increase in the polarizability and its anisotropy with respect to that of the ground state. Since the excitation energy for Br_2_ is very similar to that of Cl_2_, we assumed for Br_2_ the same change in polarizability as for Cl_2_, for which reliable values are available (Beneventi et al., 1993; Nunzi et al., 2019; Pirani et al., 2019). For the ground electronic state of Br_2_, the average polarizability value and its anisotropy have been taken from the literature (Maroulis and Makris, 1997). The interaction potential in the triplet B state has been modeled by considering exclusively the occurrence of a vdW interaction, which can be represented with a pairwise additive approach. The potential parameters (*r*_m_ , ε) have been estimated on the basis of polarizability and correlation formulas (Cambi et al., 1991).

The estimated parameters for the excited B state are reported in Table 1. For the He–Br_2_ system, the relative anisotropy of the present PES for the B state (i.e., the difference between perpendicular and parallel configuration divided by the average value) is in good agreement with that obtained by *ab initio* calculations (de Lara-Castells et al., 2004; Garcia-Vela, 2005).

In conclusion, we have found, trough a detailed experimental investigation, that the noble gas–Br adducts are affected by a selective emergence of the intermolecular halogen bond, as recently demonstrated for the companion noble gas–Cl_2_ systems (Nunzi et al., 2019; Pirani et al., 2019). In particular, it has been confirmed that for these systems, XB's peculiar effect comes into play only in the collinear configuration of the ground-state PES. The obtained results extend the phenomenology and knowledge of the intriguing weak XB.

The present study also provided a simple and accurate analytical formulation of the PESs, describing the intermolecular interaction both in the ground X and in the excited electronic B state of Br_2_. The PES has been represented as a combination of three basic interaction components, each one modulated by few and well-defined physical parameters. The model potential provided the force fields in the full space of the relative configurations and is then suitable for molecular dynamics simulations of fundamental phenomena, such as energy transfer processes.

## Author Contributions

DC and FP contributed conception and design of the study. DC, FP, and SF conducted data collection and analysis. DC wrote the first draft of the manuscript. DC and FP wrote sections of the manuscript. All the authors contributed to the development of the experimental facility and analysis instruments. All authors contributed to manuscript revision, read and approved the submitted version.

### Conflict of Interest Statement

The authors declare that the research was conducted in the absence of any commercial or financial relationships that could be construed as a potential conflict of interest.
