# Supplementary material for: Molecular Beam Scattering Experiments as a Sensitive Probe of the Interaction in Bromine–Noble Gas Complexes
Source: Front Chem. 2019 May 17;7:320. doi: 10.3389/fchem.2019.00320 (PMC6534045; doi:10.3389/fchem.2019.00320)
Supplement: Supplementary file 1 [file Data_Sheet_1.PDF]

Molecular beam scattering experiments as a sensitive probe of the interaction in bromine-noble gas complexes

David Cappelletti[1,*], Antonio Cinti[1], Andrea Nicoziani[1], Stefano Falcinelli[2] and Fernando Pirani[1]

[1]*Dipartimento di Chimica, Biologia e Biotecnologie, Università degli Studi di Perugia, via Elce di Sotto 8, 06123 Perugia, Italy*

[2]*Dipartimento di Ingegneria Civile ed Ambientale, Università degli Studi di Perugia, via G. Duranti 93, 06215 Perugia, Italy*

*corresponding author: david.cappelletti@unipg.it

Molecular beam scattering experiments as a sensitive probe of the interaction in bromine-noble gas complexes

David Cappelletti, Antonio Cinti, Andrea Nicoziani, Stefano Falcinelli and Fernando Pirani

SUPPLEMENTARY MATERIAL

As anticipated in the previous sections, in the thermal collision energy range the average component of $Q(v)$ and the oscillatory pattern provide complementary information on the intermolecular interaction, namely on the strength of the long range attraction and on the features of the well depth, respectively. Moreover, under the used conditions, all collision events occur in the thermal energy range, involve molecular partners rotating sufficiently fast respect to the average collision time and their dynamics can be treated semi-classically. The adopted treatment applies properly to collisions of elastic type, dominant at intermediate and large impact parameter (the classical equivalent of the orbital angular momentum), such as those mainly probed by the present experiments, with no loss

of any relevant information on the intermolecular interaction. Motivations of the adopted treatment mostly arise from the experimental observation in all cases of well resolved glory patterns, that suggests the occurrence of a collision dynamics mainly driven by a substantially isotropic central field. However, the interaction anisotropy must be explicitly taken into account when its role becomes more effective, generating a partial or total “glory" amplitude quenching, and this occurs when the collision velocity overcomes the value for which the collision time becomes significantly shorter than the average molecular rotation time. Under such conditions, the projectile interacts with the target molecule “suddenly” and the interacting complex, formed during the evolution of each collision event, tends to maintain “memory" of a limited number of specific configurations, to which it is associated a particular interaction. As previously made for similar cases (Cappelletti et al., 2002; Cappelletti et al., 2015) the collision dynamics, essential for the analysis of the experimental findings, has been confined in two different limiting regimes, emerging at low and high collision velocity, respectively. A *spherical model* has been employed to describe collisions at low velocities when the Cl_2 molecule has sufficient time to behave as isotropic partners. The scattering, mainly elastic, probes several relative configurations simultaneously and is driven by a central potential similar to the isotropic component (spherical average) of the full PES. An *anisotropic molecular model*, where the cross section is represented as a combination of independent contributions coming from all possible limiting configurations of each Ng-Cl_2 collision complex. That is, a sort of “Infinite Order Sudden" approximation is applied (see for instance Refs. Bartocci et al., 2015; Cappelletti et al., 2016) . Accordingly, the two regimes selectively emerge as a function of the ratio between the mean molecular rotation time, (depending on the rotational temperature of Cl_2 into the scattering chamber), and the collision time, τ_{coll}, varying with the selected velocity v of Ng beam. During the analysis the Cl_2 molecules have been considered as targets rotating sufficiently fast only for collisions occurring at low v ($v \leq 0.80$ km/s). In the low velocity range, the collisions have been then considered mainly driven by an “effective” radial potential $V(R)$, related to the isotropic component of the PES. At each R it has been evaluated by

averaging the interaction over all the angular coordinates. At higher velocity (v > 2.40 km/s), *i.e.* when $\tau_{coll} < \tau_m$, (where τ_m is…) the *anisotropic molecular* regime sets in. In this case, at each v, the individual cross sections are calculated for several different configurations and properly averaged over all the angular (Θ) coordinates. To this aim, a grid of 256 configurations have been used to evaluate the average value of each $Q(v)$. The final theoretical $Q(v)$ values, to be compared with the experimental data, have been then calculated within the *spherical model* at low v and according to the *anisotropic molecular model* at high v. At intermediate v, the switch between the dynamics of the *spherical model* and that of the *anisotropic molecular* model has been obtained as a weighted sum, depending on v, of the cross section contributions evaluate within the two different dynamical regimes, according to the procedure detailed in Refs (Bartocci et al., 2015; Cappelletti et al., 2016).
